# Erector spinae plane block in chronic pain: a retrospective study

**DOI:** 10.55730/1300-0144.5449

**Published:** 2022-04-05

**Authors:** Hanzade Aybüke ÜNAL ARTIK, Türkan ÖRNEK GÜLPINAR, Adem HALİS, Nalan ÇELEBİ

**Affiliations:** 1 Department of Algology, Uşak University Research and Training Hospital, Uşak, Turkey; 2 Department of Obstetrics and Gynecology, Liv Hospital, Ankara, Turkey; 3 Department of Anesthesiology and Reanimation, Marmara University Pendik Research and Training Hospital, İstanbul, Turkey; 4 Department of Anesthesiology and Reanimation, Division of Algology, Faculty of Medicine, Hacettepe University, Ankara, Turkey

**Keywords:** Chronic pain, nerve block, pain management

## Abstract

Patients with various aetiology of pain who underwent erector spinae plane block at different levels were evaluated at the tertiary Algology clinic. Visual analog scale (VAS) values were recorded before the block; 30 min, two weeks, and two months after the block. Medical records of fifteen patients have been obtained. The average VAS decreased from 7 ± 1 to 5 ± 3 in the second month when compared to the values before block (p < 0.01). ESP block can be an option for chronic pain in postsurgical pain syndrome and myofascial pain management.

Erector spinae plane block (ESP) is an interfacial plane block that is performed by the application of local anaesthetic between the erector spina muscle and transverse process. ESP block takes effect by diffusing local anesthetic into musculofacial part over craniocaudal multiple vertebra levels [1]. As it can be carried out from different levels, it is used for postoperative analgesic effects in various operations [2,3]. The efficiency in chronic pains is mostly based on case reports and case series [1,4,5]. The aim of this study is to evaluate the effect of ESP block on pain severity in chronic pain due to different etiologies.

Patients undergoing ESP block due to chronic pain between February 2019 and February 2021 were reviewed retrospectively. All reported research involving “Human beings” was conducted in accordance with the principles set forth in the Helsinki Declaration 2008 and local ethical approval was obtained (2021/12-05). Written informed consent was obtained from the patients for the performance of ESP block. The ESP blocks were performed by using 18 mL of bupivacaine 0.5% and 2 mL of 8 mg dexamethasone with a 22-gauge 100-mm spinal needle (Egemen, İzmir, Turkey). The intensity of pain was evaluated by using visual analog scala (VAS) before and after the block at the 30^th^ min, 2^nd^ week, and 2^nd^ month.

The patients’ demographics, ESP block levels, and VAS values are given in Table 1. The mean VAS level is 7 ± 1 before the intervention. After the ESP block, the means are respectively determined as 2 ± 2 in the 30^th^ min, 4 ±3 in the 2^nd^ week, and 5 ± 3 in the 2^nd^ month. There was a statistically significant difference between baseline and post-block VAS score (p < 0.05). The patients who have myofascial pain consist of the ones showing no response to the trigger point injection. There has been a statistically significant decrease in myofascial pain intensity in the 2^nd^ week and 2^nd^ month compared to the before block pain level (p < 0.05) as shown in Table 2. Five of the six patients who have postsurgical chronic neuropathic pain went through gynecologic and thoracic malignancy operations. Although there has been a statistically significant decrease in pain level in the 2^nd^ week compared to the before block pain level, there has not been a statistical decrease in pain level in the 2^nd^ month (p = 0.066) (Table 2). No major complications such as nerve damage, motor block, unceasing hemorrhage, and infection were observed after the intervention.

In this study, it is found that in chronic pain, which is with no response to the medical treatment, ESP block is able to decrease the pain level. Although it is ineffective for pain due to endometrium and over malignancy, ESP block has been determined as effective in postsurgical chronic pain and myofascial pain which most patients suffer from.

In a 7-patient case series, where the effctiveness of ESP blok in postthoracotomy pain was evaluded the analgesic effect losted 2–6 weeks in 4 patients, while it losted no more than 2 days in 3 patients [4]. In our study, in 2 of the 3 patients with postthoracotomy pain, pain control has been provided for 2 months. We think that this situation is based upon the dexamethasone being used. Glucocorticoids inhibit prostaglandin synthesis and nociceptive impulse transmission in myelinated C fibers [6].

Piraccaniet al. have investigated the effectiveness of ESP block in patients with myofascial pain. They stated that ESP block is effective only in short term and it does not have a significant effect in long term [7]. Tulgar et al. Stated in their case report that they provided pain palliation respectively for 8 weeks and 3 months [8]. Besides, Yürük et al. recorded that in a prospective randomized controlled study in which ESP block effectiveness is analyzed, there has been a distinct decrease in pain for 4 weeks with ESP block compared to trigger point injection therapy. Researchers linked this finding to the fact that ESP block influences and deep nociceptors [5]. In our study, there has been a statistically significant decrease in pain in our patients for 2 months.

ESP block is an inexpensive, easily applicable, safe, and effective technique in chronic pain. We believe that ESP block may be administered in patients and before the more complicating invasive procedures.

## Figures and Tables

**Table 1:** Patients’ demographics and VAS values.

				VAS change				
Etiology		Gender	Age	ESPB level	Baseline	30 min	2 weeks	2 months
Endometrium carcinoma		F	59	T8*	9	3	8	9
Over malignancy		F	78	T9*	8	3	8	8
Myofascial pain syndrome		M	41	T12	7	3	5	5
		M	44	T12	5	0	1	1
		M	43	T6*	5	0	2	5
		M	51	T6*	7	3	4	5
		F	54	T4*	6	1	2	4
		F	34	T8	7	0	0	1
		F	46	T3*	7	0	6	7
Postsurgical pain syndrome	nephrectomy	F	19	T11	8	0	1	2
	lobectomy	F	52	T8	9	1	1	2
	lobectomy	M	58	T7	7	0	1	1
	hysterectomy	F	56	T8*	10	1	2	2
	lobectomy	M	35	T8	8	4	5	8
	hysterectomy	F	49	T8*	9	5	9	9

F: female, M: male, PSPS: postsurgical pain syndrome, ESPB: Erector spinae plane block, T: thoracic

* bilateral block

**Table 2:** VAS changes in myofascial pain and postsurgical pain syndromes.

	Myofascial pain syndrome		Postsurgical pain syndrome	
	VAS^a^	P value^b^	VAS^a^	P-value^b^
Baseline	6 ± 1		9 ± 1	
Thirty min	1 ± 1	0.017	2 ± 2	0.027
Two weeks	3 ± 2	0.018	3 ± 3	0.042
Two months	4 ± 2	0.039	4 ± 4	0.066

a: mean ± standard deviation

a mean ± standard deviation Friedman test

b Wilcoxon signed rank test

## References

[1] Forero M Rajarathinam M Adhikary SD Chin KJ Erector spinae plane block for the management of chronic shoulder pain: a casereport Canadian Journal of Anaesthesia 2018 65 3 288 293 10.1007/s12630-017-1010-1 29134518

[2] Tulgar S Ahiskalioglu A De Cassai A Gurkan Y Efficacy of bilateral erector spinae plane block in the management of pain: current insights Journal of Pain Research 2019 12 2597 2613 10.2147/JPR.S182128 31695476 PMC6717717

[3] Hamed MA Goda AS Basiony MM Fargaly OS Abdelhady MA Erector spinae plane block for postoperative analgesia in patients undergoing total abdominal hysterectomy: a randomized controlled study original study Journal of Pain Research 2019 12 1393 1398 10.2147/JPR.S196501 31118757 PMC6503185

[4] Forero M Rajarathinam M Adhikary S Chin KJ Erector spinae plane (ESP) block in the management of post thoracotomy pain syndrome: A case series Scandinavian Journal of Ppain 2017 17 325 329 10.1016/j.sjpain.2017.08.013 28919152

[5] Yürük D Akkaya ÖT Polat ÖE Alptekin HA Ultrasound-guided erector spinae plane block and trapezius muscle ınjection for myofascial pain syndrome Journal of Ultrasound in Medicine 2022 41 1 185 191 10.1002/jum.15694 33713473

[6] Moeen SM Ramadan IK Elkady HA Dexamethasone and Dexmedetomidine as an Adjuvant to Intraarticular Bupivacaine for Postoperative Pain Relief in Knee Arthroscopic Surgery: A Randomized Trial Pain Physician 2017 20 7 671 680 29149146

[7] Piraccini E Calli M Taddei S Byrne H Rocchi M Erector spinae plane block for myofascial pain syndrome: only a short-term relief? Minerva Anestesiologica 2020 86 888 890 10.23736/S0375-9393.20.14523-1 32449341

[8] Tulgar S Thomas DT Suslu H Ultrasound guided erector spinae plane block relieves lower cervical and interscapular myofascial pain, a new indication Journal of Clinical Anesthesia 2019 53 74 10.1016/j.jclinane.2018.10.008 30343227

